# Immunodetectable cyclin D _1_ is associated with oestrogen receptor but not Ki67 in normal, cancerous and precancerous breast lesions

**DOI:** 10.1054/bjoc.2001.1705

**Published:** 2001-04

**Authors:** B S Shoker, C Jarvis, M P A Davies, M Iqbal, D R Sibson, J P Sloane

**Affiliations:** Dept. of Pathology, Royal Liverpool University Hospital, Duncan Building, Prescot Street, Liverpool, L69 3GA; Clatterbridge Cancer Research Trust, J.K. Douglas Laboratories, Clatterbridge Hospital, Bebington, Wirral, CH63 4JY, UK

**Keywords:** breast cancer, benign breast, cyclin D _1_, Ki67, oestrogen receptor

## Abstract

Cyclin D1 is associated with cell cycle regulation and has more recently been shown to stimulate the transcriptional functions of the oestrogen receptor (ER). Furthermore, in normal breast there is a negative association between expression of ER and the proliferation marker Ki67 indicating that either ER positive cells are non-dividing or that the receptor is down-regulated as cells enter cycle. This important relationship breaks down in many ER-positive cancers and precancerous breast lesions where the receptor is often detected on proliferating cells. The aims of the present study were to determine the interplay between ER, Ki67 and cyclin D _1_ in individual cells within the spectrum of human breast lesions ranging from normal to invasive carcinoma by using dual staining immunofluorescence. We found that in normal breast there was a strong positive association between ER and cyclin D _1_ expression. In contrast there was a strong negative association between cyclin D _1_ and Ki67 expression. Similar findings were seen for the other precancerous and cancerous breast lesions. Thus immunodetectable cyclin D _1_ within individual cells does not appear to be associated with cell cycle progression in the benign or malignant breast but instead may have important interactions with ER. © 2001 Cancer Research Campaign http://www.bjcancer.com


*British Journal of Cancer* (2001) **84**(8), 1064–1069
© 2001 Cancer Research Campaign
doi: 10.1054/ bjoc.2001.1705, available online at http://www.idealibrary.com on IDEAL®

### Immunodetectable cyclin $D_1$ is associated with oestrogen receptor but not Ki67 in normal, cancerous and precancerous breast lesions

**BS Shoker[1], C Jarvis[1], MPA Davies[2], M Iqbal[2], DR Sibson[2] and JP Sloane[1]**

[1]Dept. of Pathology, Duncan Building, Royal Liverpool University Hospital, Prescot Street, Liverpool, L69 3GA; [2]Clatterbridge Cancer Research Trust, J.K. Douglas Laboratories, Clatterbridge Hospital, Bebington, Wirral, CH63 4JY, UK

**Summary** Cyclin $D_1$ is associated with cell cycle regulation and has more recently been shown to stimulate the transcriptional functions of the oestrogen receptor (ER). Furthermore, in normal breast there is a negative association between expression of ER and the proliferation marker Ki67 indicating that either ER positive cells are non-dividing or that the receptor is down-regulated as cells enter cycle. This important relationship breaks down in many ER-positive cancers and precancerous breast lesions where the receptor is often detected on proliferating cells. The aims of the present study were to determine the interplay between ER, Ki67 and cyclin $D_1$ in individual cells within the spectrum of human breast lesions ranging from normal to invasive carcinoma by using dual staining immunofluorescence. We found that in normal breast there was a strong positive association between ER and cyclin $D_1$ expression. In contrast there was a strong negative association between cyclin $D_1$ and Ki67 expression. Similar findings were seen for the other precancerous and cancerous breast lesions. Thus immunodetectable cyclin $D_1$ within individual cells does not appear to be associated with cell cycle progression in the benign or malignant breast but instead may have important interactions with ER. © 2001 Cancer Research Campaign http://www.bjcancer.com

**Keywords**: breast cancer; benign breast; cyclin $D_1$; Ki67; oestrogen receptor

The cyclins are a family of nuclear proteins that play an important role in the control of the cell cycle. The cyclin $D_1$ gene is located on chromosome 11q13. It is amplified in approximately 15–20% of breast carcinomas whilst overexpression of the protein occurs in approximately 50% of cases (Barnes, 1997). Overexpression of the cyclin $D_1$ protein has also been demonstrated within the in situ proliferations using immunohistochemistry with the percentage of positive lesions and/or percentage of cyclin $D_1$ positive cells increasing with increasing cytological atypia (Alle et al, 1998; Gillett et al, 1998; Mommers et al, 1998; Zhu et al, 1998). The expression in cancers of cyclin $D_1$ is strongly related to the oestrogen receptor alpha (ER) status of the tumour. Oestrogens can increase the level of cyclin $D_1$ protein in early to mid $G_1$, and thus stimulate proliferation of cancer cell lines (Sutherland et al, 1995). In the normal breast, however, there is a negative association between expression of ER and the proliferation marker Ki67 indicating that either ER-positive cells are non-dividing or that the receptor is down-regulated as cells enter cycle. This important relationship breaks down in many ER-positive cancers and precancerous breast lesions where the receptor is often detected on proliferating cells (Clarke et al, 1997; Shoker et al, 1999a, 2000). Furthermore, it has been shown that D-type cyclins may intervene in activities of transcription factors through mechanisms independent of cyclin-dependent kinases. Cyclin $D_1$ can associate physically with the ER and stimulate its transcriptional functions in the absence of oestrogen (Zwijsen et al, 1997). The relationship between ER, cyclin $D_1$ and proliferation in neoplastic and non-neoplastic breast cells is thus not clear and it is possible that the normal relationship breaks down during malignant transformation. The aim of the present study was to determine the interplay between ER, Ki67 and cyclin $D_1$ within individual cells in the spectrum of breast lesions ranging from normal to invasive carcinoma by using dual staining immunofluorescence.

*Received 1 November 2000*
*Revised 11 January 2001*
*Accepted 11 January 2001*

*Correspondence to:* BS Shoker

### METHODS

### Patients

Blocks and slides from 166 patients with breast disease were obtained from the department of Pathology at the Royal Liverpool University Hospital. The following breast lesions were examined in these patients; 11 lactating breasts, 10 apocrine metaplasia, 9 sclerosing adenosis, 15 radial scars, 11 papillomas, 10 fibroadenomas, 10 phyllodes tumours, 14 hyperplasias of usual type (without atypia, HUT), 18 lobular in situ neoplasia (LIN), 8 atypical ductal hyperplasias (ADH), 11 ductal carcinoma in situ (DCIS) of low nuclear grade (LNG), 12 of intermediate nuclear grade (ING), 11 of ER-negative high nuclear grade (HNG), 9 ER-positive HNG, 10 ER-negative infiltrating ductal carcinoma (IDC) and 10 ER-positive IDC. Some of the breast samples examined contained more than one lesion. All the diagnoses were made following the Pathology Guidelines of the European and NHS Breast Screening Programmes (National Co-ordinating Group for Breast Screening Pathology, 1997). The cases of ADH were from biopsies showing benign changes only. The criteria for diagnosing ADH were those of Page and Rogers (1992). Initially, the lobular in situ proliferations were subclassified into lobular carcinoma in situ and atypical lobular hyperplasia but there were often significant difficulties in distinguishing the 2 processes which frequently merged in the same sections. Furthermore, the results eventually obtained were similar in the 2 lesions. The mean ages of the patients are given in Table 1. The menopausal status or stage of menstrual cycle for each patient was not known.

### Mitotic counting in breast cancers

The number of mitoses within 10 high powered fields (hpf, magnification × 400, field diameter 640 μm) was calculated for all the invasive breast cancers. Mitotic figures were recognized according to the criteria described by Baak and Oort (1983). In addition the mitotic activity index (MAI) was calculated and expressed as the number of mitoses per 1000 cells. These were then correlated with the percentage of cyclin $D_1$ and Ki67-positive cells within the cancers.

### Dual immunofluorescence immunohistochemistry

Dual immunofluorescent immunohistochemistry was performed by combining a primary monoclonal mouse antibody with a primary polyclonal rabbit antibody. The monoclonal antibodies used were ER 1D5 (Dako, Cambridge, UK) and cyclin $D_1$ (Dako) and the polyclonal antibodies were Ki67 (Novacastra, Newcastle upon Tyne, UK) and ER (Santa Cruz Biotechnology Inc, Santa Cruz, California, USA). In order to verify that both ER antibodies were staining the same cells, dual immunofluorescence was performed with the 2 ER antibodies on normal breast tissue and on invasive carcinomas.

4 micrometer sections were cut onto 2% aminopropyltriethoxysilane (APES)-coated slides and dried overnight at 45˚C. The sections were dewaxed in 2 changes of xylene followed by 2 changes of industrial methylated spirits and then rinsed in deionized water. Pretreatment comprised microwaving for 15 min at full power in 10 mM EDTA (pH 7). A mixture of both primary antibodies (diluted in 5% bovine serum albumin [BSA]/Tris buffered saline [TBS]) was then applied for 80 min. The dilution used for ER 1D5 was 1/30, for Ki67 1: 100, for cyclin $D_1$ 1:20 and for polyclonal ER 1:30. BSA/TBS was applied to all negative controls. A mixture of both secondary antibodies, diluted in 5% BSA/TBS, was then applied for 30 min. The secondary antibodies used were TRITC-conjugated swine anti-rabbit antibody 1:50 (Dako) and biotinylated sheep anti-mouse antibody 1:100 (Amersham Life Sciences, UK). Next fluorescein-avidin conjugate (Dako) 1:100 (diluted in 5% BSA/TBS) was applied for 30 min. Washes in TBS were performed between each step. Finally the slides were immersed in a solution of 4′, 6-diamidino-2-phenylindole (Sigma, Poole, UK) at a concentration of 250 ng $ml^{-1}$ in TBS for 10 min. The slides were then coverslipped and mounted using an antifading medium (Vectashield, Vector Laboratories, UK).

### Assessment of immunostaining

Quantification of the fluorochrome-labelled cells was performed by either scoring the entire lesion or approximately 1000 cells across several representative fields (chosen using a 4′,6-diamidino-2-phenylindole filter). Each field was examined under a high power lens for the red (TRITC), green (fluorescein) and blue (4′, 6-diamidino-2-phenylindole) fluorochromes using the appropriate filters in succession to assess the presence or absence of double-labelled cells. A triple band filter in which all three fluorochromes could be seen simultaneously was used for confirmation of dual staining. If normal breast tissue was present around the benign or malignant lesions studied then it was also examined.

### Data analysis

For all normal and pathological categories the percentage of cells staining for each marker and for both were calculated. Also calculated were the percentage of double-labelled cells that would be expected if the 2 variables were independent. This was calculated by multiplying the percentage of cyclin $D_1$ and Ki67-positive cells or the percentage of cyclin $D_1$ and ER-positive cells and then dividing by 100 for each individual lesion. The actual number of dual positive cells and the number expected were then compared using the paired *t*-test. The observed/expected (O/E) ratio gives an indication of whether, in any of the lesions studied, the 2 markers were positively or negatively associated with each other and the strength of the association. In the former, values of greater than 1 would be expected and in the latter, less than 1. The data were also analysed by using Pearsons Product Moment Correlation Coefficient (PPMCC) and the Mann–Whitney and Kruskal–Wallis tests using SPSS software for Windows NT.

### RESULTS

### Comparison of monoclonal ER 1D5 and polyclonal ER antibodies

Dual staining for both ER antibodies was performed in 9 cases of normal breast and 5 IDC (3 ER-positive). A perfect positive correlation was achieved in both normal breast and IDC (PMCC $r = 1.00$, $P < 0.0001$). Of the 1920 ER-positive cells identified in normal breast using the ER 1D5 antibody, more than 99% of the cells were also positive with the polyclonal ER antibody. The converse was also true.

### Cyclin $D_1$, ER and Ki67 in normal breast

In normal breast Ki67 positive cells represented approximately 3% of the epithelial cell population whilst only 0.3% of cells contained immunodetectable cyclin $D_1$ (Table 1). Approximately 1300 Ki67-positive cells were identified in normal breast, however, none coexpressed cyclin $D_1$. The mean percentage of ER-positive cells in normal breast was 20% (samples mainly from women in the perimenopausal age group) and in these specimens approximately half of the cyclin $D_1$-positive cells coexpressed ER (Table 2). The cyclin $D_1$-positive cells were more likely to express ER than the cyclin $D_1$-negative cells (paired *t*-test for observed *vs* expected values $P = 0.04$) and were less likely to coexpress Ki67 (paired *t*-test for observed vs expected values $P < 0.0001$). In addition, the percentage of Ki67-positive cells decreased with increasing age (PPMCC, $r = –0.427$ $P < 0.0001$) whilst the percentage of cyclin $D_1$–positive cells increased with increasing age (PPMCC, $r = 0.255$ $P = 0.02$).

### Cyclin $D_1$, ER and Ki67 in invasive cancer

Invasive breast cancers had a high percentage of Ki67 positive cells (Table 1), the percentage was significantly higher in ER-negative than ER-positive tumours (Mann–Whitney $P = 0.01$). Cyclin $D_1$-positive cells were found in 70% of ER-positive IDC and

© 2001 Cancer Research Campaign

**Table 1** The relationship between cyclin $D_1$ and Ki67 in normal, benign and malignant breast lesions

| | No of cases | Mean age (SD) | Mean no. cells counted (SD) | Mean percentage cells cyclin $D_1$ positive (SD) | Mean percentage cells Ki67 positive (SD) | Mean percentage cells dual positive (SD) | Mean percentage of dual positive cells expected (SD) | Mean observed/ expected (SD) |
|---|---|---|---|---|---|---|---|---|
| Normal breast | 81 | 48 (14) | 857 (311) | 0.30 (0.50) | 3.2 (4.7) | 0.00 (0.0) | 0.01 (0.02) | 0.0 (0.0) |
| Lactating breast | 11 | 31 (7) | 978 (235) | 0.03 (0.06) | 2.6 (2.2) | 0.02 (0.06) | 0.002 (0.004) | 6.65 (9.4) |
| Apocrine metaplasia | 10 | 55 (11) | 819 (275) | 2.7 (6.0) | 2.6 (2.2) | 0.10 (0.33) | 0.13 (0.30) | 0.27 (0.54) |
| Sclerosing adenosis | 9 | 51 (13) | 846 (311) | 1.7 (2.9) | 2.8 (2.9) | 0.03 (0.09) | 0.10 (0.19) | 0.24 (0.59) |
| Radial scar | 15 | 57 (9) | 1509 (513) | 0.72 (1.0) | 2.0 (2.2) | 0.01 (0.05) | 0.02 (0.04) | 0.19 (0.54) |
| Papilloma | 11 | 53 (12) | 1166 (109) | 4.4 (4.6) | 6.3 (3.6) | 0.10 (0.14) | 0.30 (0.40) | 0.28 (0.41) |
| Fibroadenoma | 10 | 37 (11) | 1063 (51) | 7.7 (7.6) | 9.5 (7.5) | 0.34 (0.51) | 0.98 (1.3) | 0.18 (0.20) |
| Phyllodes tumour | 10 | 53 (14) | 1090 (65) | 2.6 (3.2) | 5.1 (4.0) | 0.03 (0.06) | 0.11 (0.14) | 0.14 (0.21) |
| Hyperplasia without atypia | 14 | 53 (13) | 686 (414) | 2.7 (3.7) | 3.5 (3.6) | 0.03 (0.06) | 0.10 (0.15) | 0.31 (0.50) |
| Atypical ductal hyperplasia | 8 | 50 (9) | 829 (347) | 17 (21) | 6.0 (1.9) | 0.46 (0.97) | 1.15 (1.36) | 0.16 (0.29) |
| Lobular in situ neoplasia | 18 | 54 (8) | 791 (414) | 8.4 (13) | 2.7 (2.6) | 0.14 (0.26) | 0.32 (0.64) | 0.63 (1.0) |
| DCIS Low nuclear grade | 11 | 63 (11) | 1144 (152) | 15 (14) | 8.3 (6.3) | 0.33 (0.67) | 1.5 (2.0) | 0.13 (0.16) |
| DCIS Intermediate nuclear grade | 12 | 54 (9) | 1115 (157) | 12 (18) | 11 (10) | 0.72 (1.8) | 1.9 (4.9) | 0.27 (0.40) |
| DCIS High nuclear grade (ER negative) | 10 | 56 (13) | 866 (295) | 3.6 (4.9) | 15 (7.0) | 0.12 (0.19) | 0.60 (0.96) | 0.36 (0.51) |
| DCIS High nuclear grade (ER positive) | 9 | 58 (6) | 921 (194) | 14 (17) | 19 (15) | 0.41 (0.51) | 2.4 (3.0) | 0.21 (0.40) |
| Invasive ductal carcinoma (ER negative) | 10 | 59 (12) | 1187 (109) | 1.4 (3.5) | 44 (30) | 0.00 (0.00) | 0.26 (0.54) | 0.00 (0.00) |
| Invasive ductal carcinoma (ER positive) | 10 | 54 (12) | 1170 (164) | 10 (16) | 16 (13) | 0.25 (0.55) | 1.6 (2.6) | 0.07 (0.13) |

in 30% of ER-negative IDC. However, the number of cases that were considered positive for cyclin $D_1$ depended upon the cut-off point used (Table 3). The mean percentage of cyclin $D_1$-positive cells was significantly higher than that seen in normal breast tissue for ER-positive (Mann–Whitney $P = 0.004$) but not for ER-negative (Mann–Whitney, $P = 0.9$) breast cancers. However, no cells coexpressing cyclin $D_1$ and Ki67 were seen in ER-negative IDC and only in 2 cases (20%) of ER-positive IDC were cells coexpressing cyclin $D_1$ and Ki67 identified, although even in these cases the majority of cyclin $D_1$ cells did not coexpress Ki67. In contrast, there was a strong positive correlation between the percentage of cyclin $D_1$-positive and ER-positive cells (PPMCC, $r = 0.617$, $P = 0.0004$).

### Mitoses and their relationship to Ki67 and cyclin $D_1$ in invasive cancer

A mean of 2033 (SD 765) cells were counted for each case with a mean MAI of 7.7. The median number of mitoses per 10 hpf was 18. The MAI and the number of mitoses per 10 hpf showed a strong correlation with the percentage of Ki67-positive cells (PPMCC, $r = 0.669$, $P = 0.001$ and $r = 0.562$, $P = 0.01$, respectively). However, no significant correlation was found between the MAI or the number of mitoses per 10 hpf and the percentage of cells expressing cyclin $D_1$ (PMCC $r = –0.251$, $P = 0.3$ and $r = –0.252$, $P = 0.3$, respectively).

### Cyclin $D_1$ and Ki67 within proliferative breast disease

### *Hyperplasia without atypia*

HUT had a significantly higher mean percentage of cyclin $D_1$-positive cells than normal breast (Mann–Whitney, $P = 0.03$) and a higher mean percentage of cells coexpressing Ki67 and cyclin $D_1$ (Mann–Whitney, $P < 0.0001$) although the mean percentage of Ki67-positive cells was similar (Mann–Whitney $P = 0.23$, Table 1). However, HUT had a lower mean percentage of cyclin $D_1$-positive cells than ADH and DCIS LNG (Mann–Whitney, highest $P = 0.009$). HUT also had a lower mean percentage of Ki67-positive cells than that seen in ADH and all grades of DCIS (Mann–Whitney, highest $P = 0.04$). Of the 247 cyclin $D_1$-positive cells identified in all HUT only 4 cells coexpressed Ki67.

**Table 2** The relationship between cyclin $D_1$ and ER in normal breast and invasive carcinoma

| | No. of cases | Mean age (SD) | Mean No. cells counted (SD) | Mean percentage cells cyclin $D_1$ positive (SD) | Mean percentage cells ER positive (SD) | Mean percentage cells dual positive (SD) | Mean percentage of dual positive cells expected (SD) | Mean observed/ expected (SD) |
|---|---|---|---|---|---|---|---|---|
| Normal breast | 16 | 52 (16) | 760 (278) | 0.61 (0.65) | 20 (21) | 0.36 (0.61) | 0.15 (0.29) | 2.2 (2.0) |
| Infiltrating ductal carcinoma ER+ | 10 | 54 (12) | 1088 (86) | 12 (16) | 65 (23) | 11 (15) | 9.5 (13) | 1.0 (0.60) |
| Infiltrating ductal carcinoma ER– | 10 | 59 (12) | 1075 (185) | 1.1 (3.2) | 0.0 (0.0) | 0.0 (0.0) | 0.0 (0.0) | – |

 © 2001 Cancer Research Campaign

**Table 3** Cyclin $D_1$ positivity in breast lesions using different cut off points and their relationship to cases that contain cells coexpressing ER and Ki67

| | No. of cases | Cases with >1% cyclin $D_1$ positive cells (percentage) | Cases with >5% cyclin $D_1$ positive cells (percentage) | Cases with >10% cyclin $D_1$ positive cells (percentage) | Cases containing cyclin $D_1$+ /Ki67+ cells (percentage) |
|---|---|---|---|---|---|
| Normal breast | 81 | 6 (7.4) | 0 (0) | 0 (0) | 0 (0) |
| Lactating breast | 11 | 0 (0) | 0 (0) | 0 (0) | 1 (9) |
| Apocrine metaplasia | 10 | 3 (30) | 1 (10) | 1 (10) | 1 (10) |
| Sclerosing adenosis | 9 | 3 (33) | 1 (11) | 0 (0) | 1 (11) |
| Radial scar | 15 | 5 (33) | 0 (0) | 0 (0) | 1 (7) |
| Papilloma | 11 | 9 (82) | 3 (27) | 3 (27) | 5 (45) |
| Fibroadenoma | 10 | 8 (80) | 5 (50) | 4 (40) | 5 (50) |
| Phyllodes tumour | 10 | 5 (50) | 1 (10) | 0 (0) | 2 (20) |
| Hyperplasia without atypia | 14 | 6 (43) | 3 (21) | 0 (0) | 2 (14) |
| Atypical ductal hyperplasia | 8 | 7 (87) | 5 (62) | 5 (62) | 3 (37) |
| Lobular in situ neoplasia | 18 | 10 (56) | 8 (44) | 5 (28) | 7 (39) |
| DCIS Low nuclear grade | 11 | 9 (82) | 8 (73) | 7 (64) | 6 (55) |
| DCIS Intermediate nuclear grade | 12 | 8 (67) | 6 (50) | 4 (33) | 4 (33) |
| DCIS High nuclear grade (ER negative) | 11 | 4 (36) | 4 (36) | 2 (18) | 4 (36) |
| DCIS High nuclear grade (ER positive) | 9 | 7 (78) | 4 (44) | 4 (44) | 5 (56) |
| Invasive ductal carcinoma (ER negative) | 10 | 2 (20) | 1 (10) | 1 (10) | 0 (0) |
| Invasive ductal carcinoma (ER positive) | 10 | 5 (50) | 4 (40) | 3 (30) | 2 (20) |

### *Atypical hyperplasia and in situ neoplasia*

The mean percentage of both cyclin $D_1$-positive and Ki67-positive cells within ADH and LNG DCIS were similar and were higher than that seen in normal breast (Mann–Whitney, highest $P = 0.002$) but similar to that seen in ING DCIS and ER-positive HNG DCIS. However, ER-negative HNG DCIS, when compared with ADH and LNG DCIS, had a lower value for the mean percentage of cyclin $D_1$-positive cells (Mann–Whitney, highest $P = 0.01$) and a higher value for the mean percentage Ki67-positive cells (Mann Whitney, highest $P = 0.04$, Table 1). Only 3 cases of ADH contained any cells coexpressing cyclin $D_1$ and Ki67, representing 13 cells of a total of 885 cyclin $D_1$-positive cells counted. LIN had a higher mean percentage of cyclin $D_1$-positive cells than normal breast but showed no difference when compared with HUT, ADH and DCIS (all nuclear grades). The mean percentage of Ki67-positive cells was similar to that seen in normal breast and HUT but was lower than that seen in ADH or DCIS (all nuclear grades). Cells coexpressing cyclin $D_1$ and Ki67 were infrequently identified in all these lesions (Table 1, Table 3).

### *Other benign breast lesions*

The other benign breast lesions associated with an increased risk of 1.5–2.0 of subsequently developing breast cancer e.g. sclerosing adenosis, radial scar, papilloma and fibroadenoma, had a wide range of values for the mean percentage of cyclin $D_1$- and Ki67-positive cells (Table 1). Some of the lesions had values similar to normal breast, others similar to HUT and yet still others with values similar to ADH. However, in all breast lesions studied, cells coexpressing cyclin $D_1$ and Ki67 were either not present or only present very infrequently (Table 1, Table 3). The mean observed /expected value for all lesions, except lactating breast, was below 1 (Figure 1). The high observed/expected ratio noted in lactating breast was because only 3 cyclin $D_1$-positive cells were identified of which 2 coexpressed Ki67.

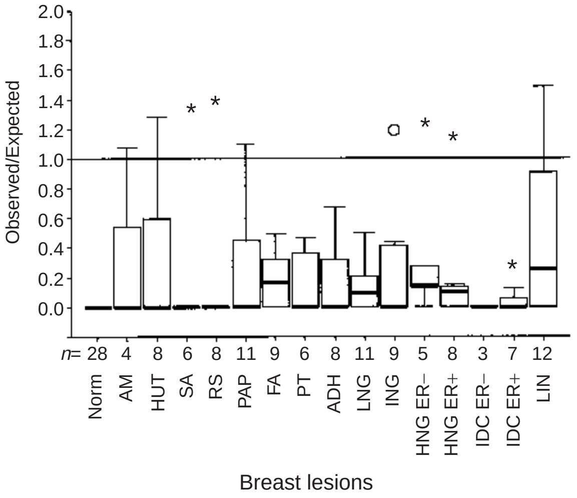

**Figure 1** Boxplot graph of the observed/expected percentage of cells coexpressing cyclin $D_1$ and Ki67 in normal breast, benign breast lesions and invasive breast carcinoma. Boxplot graphs in which the line across the box indicates the median, the box contains the values between the 25th and 75th percentiles, the whiskers extend to the highest and lowest values excluding outliers, o and * identifies outliers and extreme values (Norm, normal breast; AM, apocrine metaplasia; HUT, hyperplasia of usual type (without atypia); SA, sclerosing adenosis; RS, radial scar; PAP, papilloma, FA, fibroadenoma; PT, phyllodes tumour; ADH, atypical ductal hyperplasia; LNG, low nuclear grade ductal carcinoma in situ (DCIS); ING, intermediate nuclear grade DCIS; HNG, high nuclear grade DCIS, ER–, oestrogen receptor negative, ER+, oestrogen receptor positive; IDC, infiltrating ductal carcinoma; LIN, lobular in situ neoplasia)

### DISCUSSION

Cyclin $D_1$ is known to be important in cell cycle control by regulating progression through the $G_1$ phase of the cell cycle (Sutherland et al, 1995). Ki67 is a marker of proliferation and in the present study correlated strongly with the mitotic count and the MAI. Ki67 has also been shown to correlate with radioactive

© 2001 Cancer Research Campaign 

thymidine labelling when used as a cell cycle marker (Clarke et al, 1997). However, interestingly we did not see any Ki67-positive cells coexpressing cyclin $D_1$ in normal breast despite looking at over 1300 Ki67-positive cells. In breast cancer cyclin $D_1$ overexpression by immunohistochemistry is associated with low-grade ER-positive breast cancers that have a low proliferation rate (van Diest et al, 1997). In our study, in ER-negative breast cancers that contained a high percentage of Ki67-positive cells few or no cyclin $D_1$-positive cells were detected. Cyclin $D_1$-positive cells that were present did not coexpress Ki67. De Jong et al (1999) used dual staining immunofluorescence for cyclin $D_1$ and Ki67 on 6 cancers, 3 of which were from the breast. Coexpression of cyclin $D_1$ and Ki67 was not seen in the breast cancers but 2 squamous cell carcinomas from the head and neck region contained occasional dual-labelled cells in the basal layers. Although co-expression of cyclin $D_1$ and Ki67 is occasionally seen within the in situ proliferations, cyclin $D_1$-positive cells generally are less likely to be dividing than the cyclin $D_1$-negative cells. Hence in the majority of cases, it would appear that cyclin $D_1$ expression is not associated with cell cycle progression.

In normal breast we found that cyclin $D_1$ and ER were positively associated. Furthermore, the percentage of cyclin $D_1$-positive cells increased with age, a similar effect was seen for ER in normal breast (Shoker et al, 1999b). This supports the finding that cyclin $D_1$ is an ER-regulated gene (Sutherland et al, 1995), possibly being more likely to be switched on in environments in which low serum oestrogen concentrations prevail allowing cells to differentiate along an ER-positive pathway. Cyclin $D_1$-positive cells were also more likely to be present within ER-positive than ER-negative DCIS and invasive cancer. The disparity between the amplification of the cyclin $D_1$ gene and the overexpression of cyclin $D_1$ protein in breast cancers (Barnes, 1997) may therefore be due to the dysregulation of ER that occurs in the majority of these lesions (Shoker et al, 1999a, 1999b, 2000). The mutual expression of cyclin $D_1$ and ER could allow the direct stimulation of ER pathways in the absence of oestrogen and may have an important role in the aetiology of ER positive cancers (Zwijsen et al, 1997).

The overexpression of the cyclin $D_1$ gene has been associated with high telomerase activity without an increase in tumour cell proliferation (Landberg et al 1997). In a mammary epithelial cell line, transduction of cyclin $D_1$ inhibited growth, possibly due to prolongation of the S-phase (Han et al, 1995). Similarly, expression of ER in previously ER-negative cell lines leads to growth inhibition (Zajchowski et al, 1993). The prevention of ER or cyclin $D_1$-expressing cells to enter division is possibly achieved by the action of cyclin-dependent kinase inhibitors, e.g. $p21^{Cip1}$ and $p27^{Kip1}$, which have been described as markers of differentiation in breast epithelia. Cells overexpressing cyclin $D_1$ also frequently coexpress $p21^{Cip1}$ (de Jong et al, 1999) and, in grade I breast cancers, $p27^{Kip1}$ expression is correlated with both ER and cyclin $D_1$ (Leong et al, 2000). Whilst both ER and cyclin $D_1$ play an important role in cellular proliferation and in tumorigenesis, their action would seem to favour differentiation and thus lead to an association with less aggressive breast tumours. The expression of cyclin $D_1$ may therefore be important in both proliferation and differentiation, the pathway followed depending upon other cell cycle regulators that are also present within the cell.

Overexpression of the cyclin $D_1$ protein is common in breast cancer (Barnes, 1997). In transgenic mice, when the cyclin $D_1$ gene is linked to the mouse mammary tumour virus promoter, the mice develop precancerous hyperplasias and only after long latent periods do they develop carcinomas (Wang et al, 1994). Thus a number of studies have looked at precancerous breast lesions to see whether this represents an early change in the progression to neoplasia. One of the first studies looked at cyclin $D_1$ mRNA expression using in situ hybridization. It found overexpression in 18% of benign breast lesions and ADH and in 76% of LNG DCIS, 87% HNG DCIS and 83% IDC and suggested that cyclin $D_1$ may be useful in separating benign and premalignant breast lesions from any form of breast carcinoma (Weinstat-Saslow et al, 1995). Gillett et al (1998) looked at protein overexpression by immunohistochemistry and found that it was present in 64% of cases of DCIS and in only 14% of cases of ADH although a further 7 of 9 cases did show weak staining. A number of other studies have found a graded increase in cyclin $D_1$ overexpression from normal to HUT to ADH to DCIS and finally to invasive cancer with significant differences not always being seen between the lesions (Alle et al, 1998; Mommers et al, 1998; Zhu et al, 1998). Our own findings agree with these latter studies. The mean percentage of cyclin $D_1$-positive cells is higher within the in situ proliferations than in normal breast. ADH had values higher than those seen for HUT but similar to those seen in ER-positive DCIS. The percentage of cyclin $D_1$-positive cells in these lesions thus seems to correlate with the percentage of ER-positive cells also found in these lesions (Shoker et al, 1999a, 1999b, 2000). However, the other low-risk breast lesions contain a variable percentage of cyclin $D_1$-positive cells, some lesions have similar values to HUT whilst other have higher values that approach the atypical proliferations. The usefulness of cyclin $D_1$ in separating benign from malignant lesions is therefore not clear cut.

### ACKNOWLEDGEMENT

BSS was funded by a grant from the Cancer Research & Polio Fund

### REFERENCES

Alle KM, Henshall SM, Field AS and Sutherland RL (1998) Cyclin D1 protein is overexpressed in hyperplasia and intraductal carcinoma of the breast. *Clin Cancer Res* **4**: 847–854

Baak JPA and Oort J (1983) *A manual of morphometry in diagnostic pathology* Springer: Heidelberg, Berlin, New York

Barnes DM (1997) Cyclin $D_1$ in mammary carcinoma. *J Pathol* **181**: 267–269

Clarke RB, Howell A, Potten CS and Anderson E (1997) Dissociation between steroid receptor expression and cell proliferation in the human breast. *Cancer Res* **57**: 4987–4991

de Jong JS, van Diest PJ, Michalides RJAM and Baak JPA (1999) Concerted overexpression of the genes encoding p21 and cyclin D1 is associated with growth inhibition and differentiation in various carcinomas. *J Clin Pathol: Mol Pathol* **52**: 78–83

Gillett CE, Lee AHS, Millis RR and Barnes DM (1998) Cyclin D1 and associated proteins in mammary ductal carcinoma in situ and atypical ductal hyperplasia. *J Pathol* **184**: 396–400

Han EK, Sgambato A, Jiang W, Zhang YJ, Santella RM, Doki Y, Cacace AM, Schieren I and Weinstein IB (1995) Stable overexpression of cyclin D1 in human mammary epithelial cell line prolongs the S-phase and inhibits growth. *Oncogene* **10**: 953–961

Landberg G, Nielsen NH, Nilsson P, Emdin SO, Cajander J and Roos G (1997) Telomerase activity is associated with cell cycle deregulation in human breast cancer. *Cancer Res* **57**: 549–554

Leong AC, Hanby AM, Potts HW, Tan DS, Skilton D, Ryder K, Harris WH, Liebmann RD, Barnes DM and Gillett CE (2000) Cell cycle proteins do not predict outcome in grade I infiltrating ductal carcinoma of the breast. *Int J Cancer* **89**: 26–31

 © 2001 Cancer Research Campaign

Mommers ECM, van Diest PJ, Leonhart AM, Meijer CJLM and Baak JPA (1998) Expression of proliferation and apoptosis-related proteins in usual ductal hyperplasia of the breast. *Hum Pathol* **29**: 1539–1545

National co-ordinating group for breast screening pathology (1997) *Pathology reporting in breast cancer screening* 2nd edn. NHSBSP Publications No 3 Sheffield, UK

Page DL and Rogers LW (1992) Combined histologic and cytologic criteria for the diagnosis of mammary atypical ductal hyperplasia. *Hum Pathol* **23**: 1095–1097

Shoker BS, Jarvis C, Clarke RB, Anderson E, Hewlett J, Davies MPA, Sibson DR and Sloane JP (1999a) Oestrogen receptor- positive proliferating cells in the normal and precancerous breast. *Am J Pathol* **155**: 1811–1815

Shoker BS, Jarvis C, Sibson DR, Walker C and Sloane JP (1999b) Oestrogen receptor expression in the normal and precancerous breast. *J Pathol* **188**: 237–244

Shoker BS, Jarvis C, Clarke RB, Anderson E, Munro C, Davies MPA, Sibson DR and Sloane JP (2000) Abnormal regulation of oestrogen receptor in benign breast lesions. *J Clin Pathol* **53**: 778–783

Sutherland RL, Hamilton JA, Sweeney KJE, Watts CKW and Musgrove EA (1995) Expression and regulation of cyclin genes in breast cancer. *Acta Oncol* **34**: 651–656

van Diest PJ, Michalides RJ, Jannink L, van der Valk P, Peterse HL, de Jong JS, Meijer CJ and Baak JP (1997) Cyclin D1 expression in invasive breast cancer. Correlations and prognostic value. *Am J Pathol* **150**: 705–711

Wang TC, Cardiff RD, Zukerberg L, Lees E, Arnold A and Schmidt EV (1994) Mammary hyperplasia and carcinoma in MMTV- cyclin D1 transgenic mice. *Nature* **369**: 669–671

Weinstat-Saslow D, Merino MJ, Manrow RE, Lawerence JA, Bluth RF, Wittenbel KD, Simpson JF, Page DL and Steeg PS (1995) Overexpression of cyclin D messenger RNA distinguishes invasive and in situ breast carcinomas from non malignant lesions. *Nat Med* **1**: 1257–1260

Zajchowski DA, Sager R and Webster L (1993) Oestrogen inhibits the growth of oestrogen receptor-negative, but not oestrogen receptor-positive, human mammary epithelial cells expressing a recombinant oestrogen receptor. *Cancer Res* **53**: 5004–5011

Zhu XL, Hartwick W, Rohan T and Kandel R (1998) Cyclin D1 gene amplification and protein expression in benign breast disease and breast carcinoma. *Mod Pathol* **11**: 1082–1088

Zwijsen RML, Wientjens E, Klompmaker R, van der Sman J, Bernards R and Michalides RJAM (1997) CDK-independent activation of oestrogen receptor by cyclin D1. *Cell* **88**: 405–415

*© 2001 Cancer Research Campaign*
